# Frequency Seismic Response for EEWS Testing on Uniaxial Shaking Table

**DOI:** 10.3390/e25040655

**Published:** 2023-04-14

**Authors:** Codrin Donciu, Elena Serea, Marinel Costel Temneanu

**Affiliations:** Faculty of Electrical Engineering, “Gheorghe Asachi” Technical University of Iași, 700050 Iași, Romania; cdonciu@tuiasi.ro (C.D.);

**Keywords:** earthquake, seismic response, frequency, shaking table

## Abstract

Earthquake early warning systems are used as important tools in earthquake risk management, providing timely information to residents and both public and private emergency managers. By doing this, the potential impact of large magnitude seismic events is significantly reduced. These systems use seismic sensors in order to acquire real-time data for the weaker but fast moving P wave (usually the first 3–5 s of the earthquake) and specific algorithms to predict the magnitude and the arrival time of the slower but more destructive surface waves. Most of these projection algorithms make use only of the vertical component of the acceleration and need extensive training in earthquake simulators in order to enhance their performance. Therefore, a low-inertial-mass uniaxial shaking table is proposed and analyzed in terms of frequency response in this paper, providing an effective cost/control ratio and high daily duty cycle. Furthermore, with the large variety of prediction algorithms, which use different frequency ranges, a new concept of selective frequency band error is also introduced and discussed in this paper as being a necessary tool for the final assessment of magnitude estimation algorithm error.

## 1. Introduction

The lithospheric plates of Earth are continuously set in motion by their thermal energy through the convection currents inside the mantle, generated by the difference between the high temperatures of the nucleus and the low temperatures of the mantle. When the plates slide past one another, this energy is internally stored and when a fault interaction or rupture zone occurs, the energy is suddenly released as radiated energy, fracture energy and thermal energy [1]. The share of the potential energy that radiates propagates through the crust outward in all directions in the form of seismic (elastic) waves. Only rapidly slipping (or seismic) events generate elastic waves, while the energy released during slow-slip events (aseismic events) would mostly dissipate [2]. The radiated energy is ordinarily calculated by measuring the energy flux at the point where the wave field of an earthquake is recorded, and from that inferring the total energy flux in all directions from the epicenter [3]. For spectral models and ground motion prediction, it is assumed that earthquakes radiate about half of the available strain energy into the surrounding medium [4]. Radiation efficiency, as the ratio between seismic radiated energy and the available strain energy, is correlated with fault geometry and symmetry and with rupture velocity.

Based on the energy released, an earthquake is quantitatively mainly inferred after moment magnitude M_w_ (logarithmic scale). One magnitude corresponds to several distributed intensities, depending on the distance from the hypocenter: as distance decreases, the intensity increases [5]. Being assessed as a scattered seismic effect, earthquake intensity is a qualitative measure, which together with the direction of the fault rupture propagation and with the surface geology gives the weight of the potential damage [6]. Co-seismic, post-seismic and inter-seismic impact regarding crustal deformations, structural damage [7] and humming to the endangerment of any integrity is contingent on the approach to disaster preparedness. For the most exposed areas with the highest density and vulnerability to earthquakes, it is necessary to ensure a good resilience of the assets [8], namely non-structural or structural systems, of which the most exposed are the buildings. Each presents a degree of seismic fragility that can be effectively predicted using an analytical–mechanical-based procedure, revealing the seismic behavior of building portfolios on a typological base [9]. The seismic response can be accurately predicted for reinforced concrete buildings as well, with different degrees of diaphragm flexibility, and for non-structural components [10], facilitating the creation of a system model that is as realistic as possible in order to include the seismic effects in a local resilience plan. This new concept of resilience comprises the capabilities of a complex system, composed of interacting physical and social components, to withstand external stress and return to a state of equilibrium or bounce forward to improved new states of equilibrium [11]. These capabilities are bordered by the disaster response and community preparedness in case of an earthquake. Whereas some capabilities must be mobilized subsequently to support disaster responses, others need to serve a particular response situation continuously and accurately. A mechanism that upholds this type of capability is the earthquake early warning system (EEWS), an integrated architecture of hazard monitoring, forecasting and prediction, disaster risk assessment and communication [12]. EEWSs are critical not only in proximate areas but also far from the epicenter because earthquake magnitude and location cannot be precisely detected in the first instance [13]. Typically, earthquake parameters are estimated based on simultaneous real-time waveform records from three to six stations [14]. The detection is based on the primary P wave propagation and the EEWS trigger is settled after computing the secondary S wave travel time, proportional to the distance from the epicenter. The warning window can range from a few seconds to tens of seconds (Table 1), depending on the size of the earthquake and on the number and type of sensors in the EEWS architecture [15]. S wave arrival time is defined as the duration in which the S wave travels from the first to the farthest observation sensor from the epicenter. The arrival values are computed using point source algorithms (based on ground motion prediction-specific equations), which retrieve information on the P wave from at least three stations in the vicinity of the epicenter. The prediction algorithms neglect the tectonic stratigraphy or the depth of the earthquake and serve as an approximate guide because the shaking intensity at any specific location can vary compared to the average shaking at that distance according to ground motion prediction equations; thus, the strongest shaking can actually be felt later than the S wave arrival time [16].

Seismic sensors are the most critical elements of seismographs or EEWS, as they must measure dynamic ground motion higher than the ground noise distinctly from human-induced artificial noise. Their frequency band must cover 0.01 to 100 Hz and ground motions from 1 nm to 10 mm [18]. From classical seismic receivers placed at or close to a designated area [19] to downhole receivers immersed at typical depths [20] or to latest optical fiber network enhancing [21], seismic sensors detect certain ground vibrations with the highest precision and process the information in real-time to trigger an alert. Lately, seismic recording instruments have gained particular importance in assisting the monitoring processes of other natural phenomena, such as rainfall episodes or thunderstorms [22], or for target detection and activity recognition [23]. Seismic nodal sensors differ in terms of performance, ease of deployment, size, power consumption, data format and storage and battery lives [24], characteristics that are reflected in their cost and further in the spatial distribution and density of the earthquake monitoring networks.

Since both the sensor’s technology (range, resolution, noise density and frequency response) and prediction algorithms (including data preprocessing) are continuously evolving, researchers need an enormous volume of training data in order to match the seismic waveform parameters to the real-time (sensor-specific) acquired data and projection algorithm’s error.

In order to fulfill the increasing demand of easy-to-use but also precise earthquake simulators, a low-inertial-mass (tailored to the purpose of carrying only the seismic sensor) uniaxial shaking table (because most algorithms use only the vertical component of the acceleration) is proposed in this paper.

By maintaining the hardware arrangement to a minimum, the pre-experimental phase is lowered and therefore the proposed solution provides an effective cost/control ratio and high daily duty cycle.

Its performances, in terms of frequency response, are compared with those reported in the literature. Furthermore, a new concept of selective frequency band error is also introduced and discussed in this paper, as being a necessary tool for the final assessment of magnitude estimation error.

## 2. Laboratory Small-Scale Shaking Table Design and Control

An earthquake laboratory reproduction is better realized with six-degrees-of-freedom vibrating tables, because structures are generally excited to three orthogonal components of ground motion: two orthogonal horizontal components in the principal directions of structures (chosen for convenience of understanding and analysis) and one vertical component of the earthquake [25]. As EEWS use only the vertical component for estimating earthquake parameters (the maximum acceleration is obtained from the vertical component of the waveform [26]), the designed shaking table for EEWS sensor testing is unidirectional (single axis and single degree of freedom).

The performance evaluation of unidirectional shaking tables has been issued in much research, addressing the interaction effects with different loading configurations [27], the accuracy of a small-scale low-cost electrodynamic shaker [28] and the bearing capacity issues when reproducing sinusoidal ground motion with frequencies up to 10 Hz [29], or revealing synchronization and tracking control advances [30]. The shaking table test outlasts the seismic test closest to the real seismic response, exhaustively appraising the impact of damping natural vibration frequency in all types of investigations, from laboratory validations to practical studies for settling the dynamic behavior of a dry-joint masonry arch [31] or to evaluate the effectiveness of coupling between a 2-degree-of-freedom shear-type frame system and a rigid block [32]. Intended for simulating all types of vibrations generated by sine waves, chirp signals or scaled earthquakes, the frequency range of small electrodynamic tables is 0~10 kHz, and for wide size tables for large process models it is 0~2 kHz. Sine wave and random wave tests can be realized; the acceleration waveform distortion is small, but executing large output and large displacement tests remains difficult [33]. To determine the essential characteristics of the seismic analysis for coupled systems or nonstructural components, a small-scale vibration table with a control algorithm (PID, adaptive, neural network or fuzzy control) provides an alternative cost-effective research method [34]. Reducing the vulnerability of non-structural components (such as EEWS) is crucial for achieving full earthquake resilience and for avoiding the loss of functionality and downtime. Although EEWSs are very efficient in reducing damage and have a reliable response thanks to quality control tests, they often show a brittle behavior that may strongly reduce global robustness in the case of extreme and rare seismic actions. This highlights the need for additional studies with the purpose of the exact correlation of safety coefficients to required reliability levels [8].

**Figure 1:**
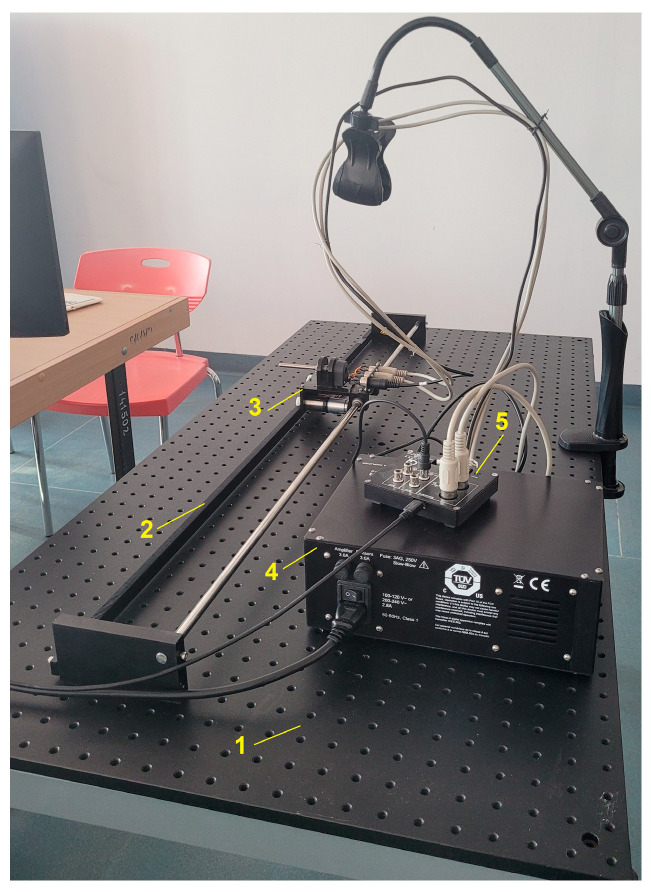
Small-scale unidirectional vibration table prototype.

The shaking table model from Figure 1, designed to test EEWS sensors, consists of three functional parts, with the following subcomponents:

- Mechanical part: (1) a standard steel plate, (2) a slide cart (linear rail system) with smooth surfaces to minimize the frictional resistance and auxiliary fixing elements.
- Electrodynamic part: (3) an actuator able to drive the mechanical component (a DC micromotor and an optical encoder), supplied through a power amplifier (4).
- Controlling part: computer with LabVIEW interface for generating input signals and (5) a data acquisition board through which command and data acquisition are executed.

**Figure 2:**
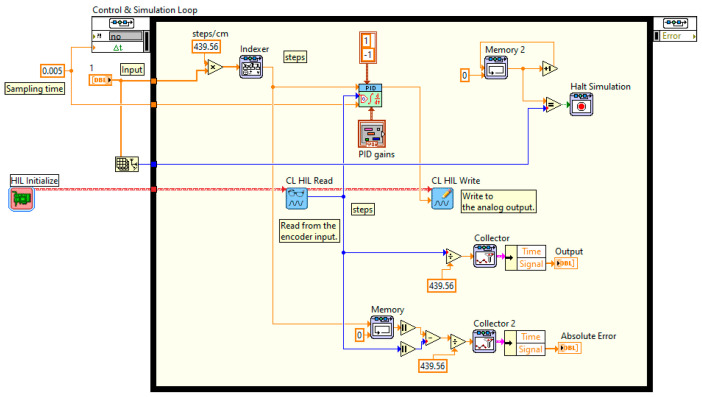
Block diagram of a unidirectional shaking table control with displacement feedback.

Usually, to control table motions in order to reproduce seismic vibration, a PID algorithm for the shake table response is used [35]. Under the PID scheme the controller responds to the difference between the table command and feedback displacements (the error). This value is continously calculated and the displacement is corrected by the controller based on predefined ratios of proportional, integral, and derivative of the error [36]. The implemented control loop (Figure 2) operates in control displacement mode and to optimize the signal reproduction by controlling feedback parameters, which are continuously adjusted to increase the precision in acceleration.

## 3. Test Results

To establish stability contingency, frequency domain analysis was performed for 12 earthquake sequences, as listed in Table 2.

These reference sequences, obtained from the PEER Ground Motion Database and available at https://ngawest2.berkeley.edu/, accessed on 17 October 2022, were selected to fulfill the following conditions:

- High enough magnitude because most of the EEWSs trigger the alarm at magnitudes higher than six;
- Different enough spectral content because the simulator’s performances are evaluated in the frequency domain.

The amplitude of a seismic wave recorded in a site depends on two parameters: the magnitude of the earthquake measured in the epicenter and Rjb—the surface distance from the epicenter to the site. In order to compare the seismic waves, they were chosen from the database records with constant Rjb (as can be seen in Table 2, between 111 and 130 km) and variable magnitude in the coverage area of the EEWS (>5.9 Mw). Moreover, to verify the reproducibility of the data, a series of earthquakes with similar Mw and Rjb, but recorded at different stations, were selected: for Chi-Chi Taiwan-02, the data from three sites (stations) were processed, and for Parkfield-02 CA, Chi-Chi Taiwan-03 and San Fernando, the data from two different sites (stations) were processed. For the purposes of being informative, and for observing the depth at which the earthquake occurred, the parameter Rrup is given together with Rjb.

**Figure 3:**
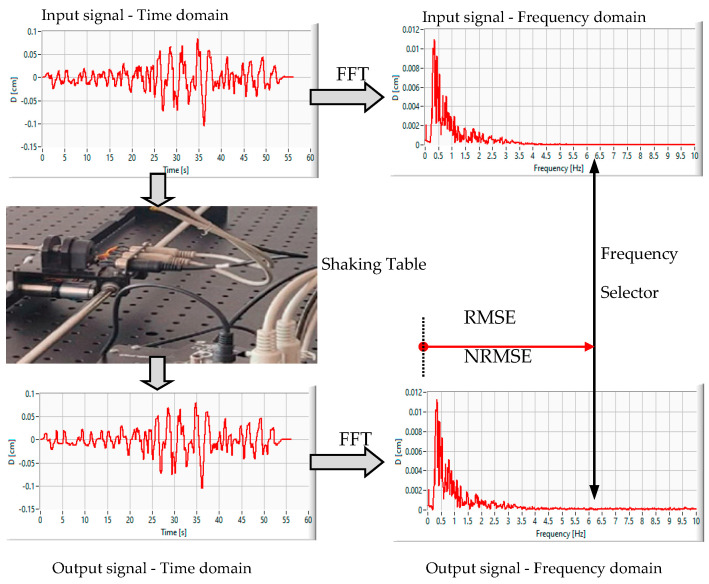
Experimental information flow: left panels: reference and output displacement waveforms and right panels: frequency spectrum with the error calculation point indicator.

The experimental information flow from Figure 3 introduces the uniaxial shaking table as a displacement tracking system feed with the time domain displacement waveforms, as selected from the PEER database. Every seismic event from the sequence of 12 is treated individually, as the sapling parameters differ (from 0.004 s to 0.005 s). Both reference and output (slide cart) displacement are analyzed in the frequency domain using fast Fourier transform to obtain the frequency spectrum.

The performance in the frequency domain of the shaking table is further evaluated from two perspectives:

1. Locally, for each individual frequency magnitude, in terms of magnitude absolute error.
2. Globally, for the entire spectrum, in terms of root mean square error and normalized root mean square error, as to be compared with other reported results.

The analysis of the results starts with the evaluation of the absolute error, a type of primary information about the system’s performances.

**Figure 4:**
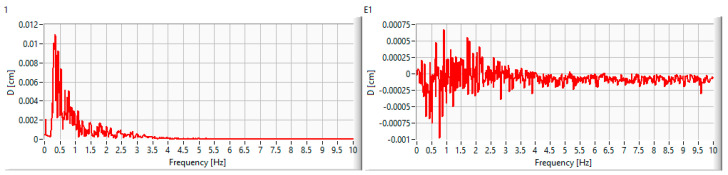

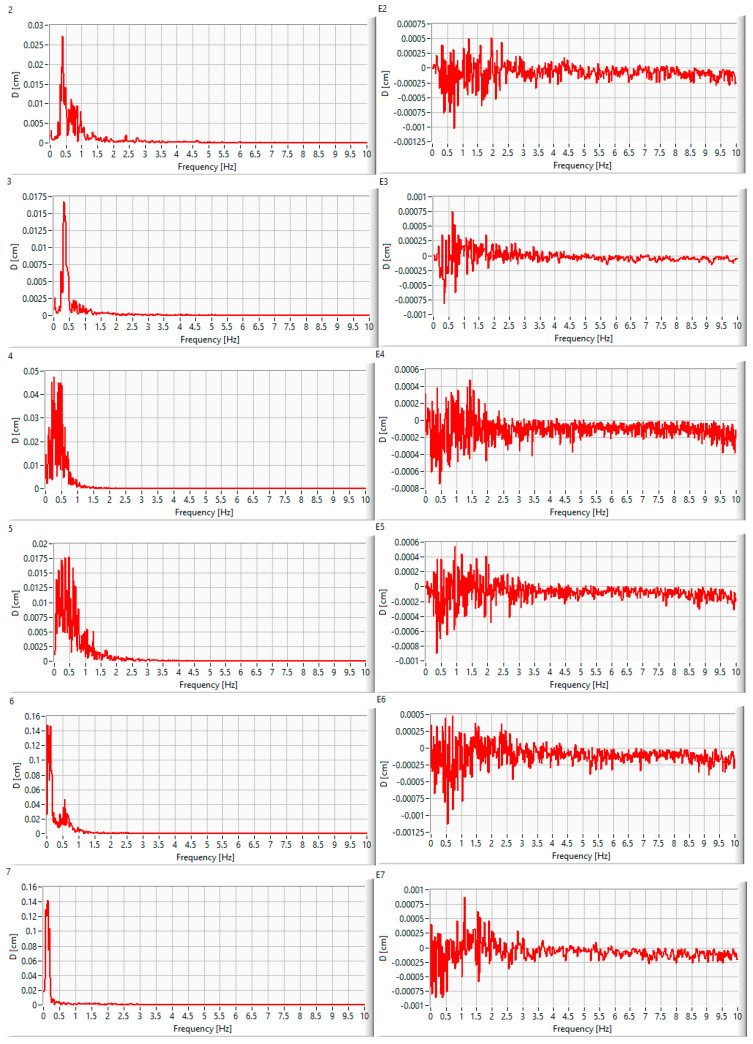

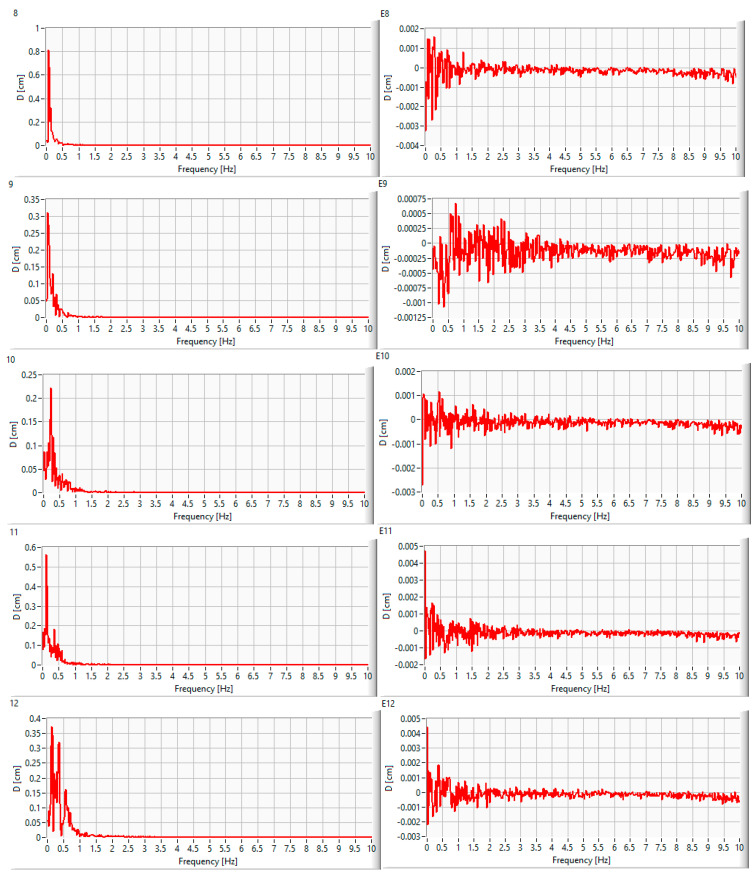
Shaking table frequency seismic response to unidirectional input ground motions ((**1**–**12**): the reference earthquake sequence; (**E1**–**E12**): the associated displacement absolute errors).

In Figure 4, spectrograms of the reference signal (left column) and the absolute error for each spectral frequency (the difference between the amplitude of the same *i* spectral component in the input *I_i_* and output *O_i_* spectrograms) are depicted. The evaluation range is from 0 to 10 Hz (the highest reachable frequency that corresponds to the natural frequency), as this is what typical seismographs routinely record [37].

From the graphs displayed in Figure 4, it can be seen that the absolute errors of the proposed shaking table model, calculated as the difference between the reference and the output frequency spectrums, are very small, with a band amplitude of 0.001 cm for small events and 0.0032 for large events.

However, the individual absolute errors do not give a relevant overview of the system’s performances, with the universally recognized indicator for analyzing clustered data being the root mean square error (*RMSE*). Therefore, in Section 4, the results are discussed using this approach for comparing the model fitting for 12 seismic response variables and for assessing residual variance.

## 4. Discussion

An important aspect of the error metrics used for simulators’ evaluation is their capability to discriminate among simulation results. Giving higher weighting to the unfavorable conditions, the root mean square error (*RMSE*) is better at revealing performance differences, being the widely used standard statistical metric that measures performance in natural phenomena studies [38]. For a specific spectrogram, with *n* points in the frequency domain, *RMSE* is defined as

$${RMSE}_{}=\sqrt{\frac{\sum_{i=0}^{n}(I_{i}-O_{i}{)}^{2}}{n}}$$

with *I_i_* being the reference seismic frequency from the database (amplitude of spectral component *i*) and *O_i_* being the recorded seismic frequency from the shaking table (amplitude of spectral component *i*).

However, the use of this global performance indicator can lead to an erroneous assessment of the quality of the prediction algorithm.

As pointed out before, all prediction algorithms use extensive sets of experimental data, but these data are preprocessed prior to feeding the computational unit. The preprocessing includes one or more bandpass filtering stage, an essential but an unstandardized process since the high cutoff frequency varies from 3 to 10 Hz. It is important to use a performance indicator tailored to the specific bandpass of each algorithm if its quality is to be assessed.

**Figure 5:**
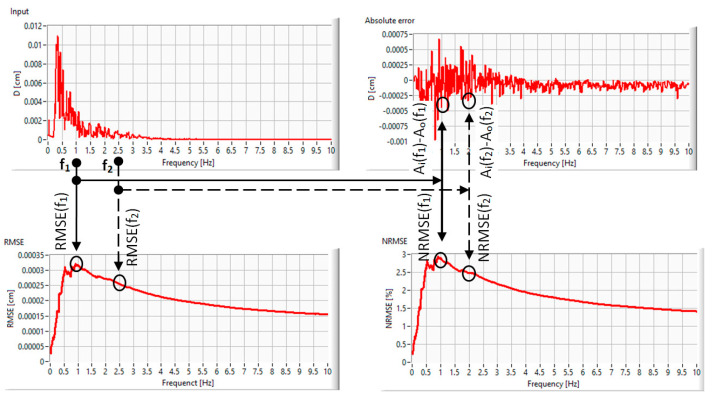
Error evaluation process using selective band frequency for two arbitrarily selected frequencies: *RMSE* (**left** panels) and *NRMSE* (**right** panels).

Therefore, we will discuss the performance of the proposed shaking table in terms of selective frequency band error, with the process being depicted in Figure 5.

For different frequencies, different values of *RMSE* are computed using only the components lower than the frequency of interest:

$${RMSE}_{f}=\sqrt{\frac{\sum_{i=0}^{\frac{f}{∆f}}(I_{i}-O_{i}{)}^{2}}{\frac{f}{∆f}}}\left[\mathrm{cm}\right]$$

where *f* = the frequency of interest, Δ*f* = the resolution in frequency and *f*/Δ*f* = sample size, with the number of measuring points in the frequency spectrum of [0, f].

Normalization was further included to assess the simulator’s performance, as it is sensitive to the amplitude and frequency errors and provides better identification of the output fitting correctness, similar to reference [39]. The normalized root mean square error was calculated using the following equation:

$$NRMSE=\frac{RMSE}{{max(I}_{i})-min(I_{i})}\left[\%\right]$$

*RMSE* and *NRMSE* variation are presented in Table 3, for four different band frequencies

The obtained results reveal two important aspects:

- Global *RMSEs*, computed for all spectral components to be compared with other reported results, have lower values than those reported in recent papers [39,40,41,42]. Even if simple control algorithms have been used, the minimization of the hardware arrangement, and the low weight of the moving parts (only 390 g for the cart and accelerometer), yields very good seismic-waveform-tracking characteristics. Additionally, the absence of some heavy actuators makes the pre-experimental phase very short, hence ensuring a high daily duty cycle.
- For lower cutoff frequencies, both *RMSE* and *NRMSE* increase their values. The assessment of the prediction algorithm accuracy by using the standard (full frequency domain) *RMSE* can lead to unrealistic expectations.

## 5. Conclusions

Research into earthquake simulation for particular vibration effects on structural and non-structural elements is mainly performed using a shaking table. It can realistically simulate the state of stress or deficiency of the tested system under a seismic action, but its dynamic behavior is poor because of the heavy moving parts and actuators. In order to perform seismic sensor testing, as required by EEWS algorithm development and optimization, a tailored uniaxial shaking table was developed and analyzed in terms of dynamic response.

Meeting a set of favorable characteristics, from minimal architecture with low-weight moving parts to a simple control algorithm, the proposed shaking table yield is effective in tracking fidelity and the cost/control ratio. Moreover, because of the absence of some heavy actuators, the pre-experimental phase is very short, ensuring a high daily duty cycle.

When analyzing the system’s frequency domain performances, it is important to use an indicator tailored to the specific bandpass of each EEWS prediction algorithm. Therefore, a new frequency domain error evaluation tool is used and defined as being appropriate for contributing to a more accurate assessment of the quality of the prediction algorithms.

## Figures and Tables

**Table 1:** Example of EEWS appraisal for seismic parameters (after [17]).

Earthquake Moment Magnitude (M_w_)	Approximate Fault Length (km)	Maximum Epicentral Distance Where Earthquake Is Expected (km)	S Wave Arrival at Maximum Distance Where Earthquake Is Expected (s)	Potential Damage
5	1	10	4	insignificant
5.9	6	40	10	minor
6	50	200	40	moderate
6.9 (crustal)	400	700	200	moderate to major
7 (subduction)	1000	1000	300	major

**Table 2:** Seismic parameters used as the reference for testing.

No.	Name	Year	Station Name	Magnitude (Mw)	Rjb (km)	Rrup (km)	Sampling Period (s)
1	Chi-Chi_Taiwan-02	1999	CHY065	5.9	125.26	125.89	0.005
2	Chi-Chi_Taiwan-02	1999	CHY067	5.9	126.39	126.56	0.004
3	Chi-Chi_Taiwan-02	1999	CHY071	5.9	122.02	122.19	0.005
4	Parkfield-02_CA	2004	Hollister—Airport Bldg #3	6	121.51	121.54	0.005
5	Parkfield-02_CA	2004	Salinas—County Hospital Gnds	6	120.74	120.79	0.005
6	Chi-Chi_Taiwan-03	1999	ILA006	6.2	129.11	129.4	0.004
7	Chi-Chi_Taiwan-03	1999	ILA007	6.2	127.25	127.54	0.004
8	San Fernando	1971	Isabella Dam (Aux Abut)	6.61	130	130.98	0.005
9	San Fernando	1971	Bakersfield—Harvey Aud	6.61	111.88	113.02	0.005
10	El Alamo	1956	El Centro Array #9	6.8	121	121.7	0.005
11	Hector Mine	1999	Bombay Beach Fire Station	7.13	120.69	120.69	0.005
12	Lander	1992	Covina—W Badillo	7.28	128.06	128.06	0.005

**Table 3:** *RMSE* and N*RMSE* values for different band frequencies.

Cutoff Frequency	*RMSE* (Cm) Min.	*RMSE* (cm) Max.	*NRMSE* (%) Min.	*NRMSE* (%) Max.
3 Hz	0.00021	0.00061	0.09	2.18
5 Hz	0.00019	0.00047	0.058	1.75
7 Hz	0.00017	0.00042	0.05	1.55
10 Hz	0.00016	0.00038	0.044	1.48

## Data Availability

Not applicable.
